# Meta-analysis across Nellore cattle populations identifies common metabolic mechanisms that regulate feed efficiency-related traits

**DOI:** 10.1186/s12864-022-08671-w

**Published:** 2022-06-07

**Authors:** Lucio F. M. Mota, Samuel W. B. Santos, Gerardo A. Fernandes Júnior, Tiago Bresolin, Maria E. Z. Mercadante, Josineudson A. V. Silva, Joslaine N. S. G. Cyrillo, Fábio M. Monteiro, Roberto Carvalheiro, Lucia G. Albuquerque

**Affiliations:** 1 School of Agricultural and Veterinarian Sciences, São Paulo State University (UNESP), Jaboticabal - SP, São Paulo, 14884-900 Brazil; 2 Institute of Animal Science, Beef Cattle Research Center, Sertãozinho - SP, São Paulo, 14174-000 Brazil; 3 National Council for Science and Technological Development, Brasilia - DF, 71605-001 Brazil; 4 School of Veterinary Medicine and Animal Science, São Paulo State University (UNESP), Botucatu – SP, 18618-681 Brazil

**Keywords:** Beef cattle, Energy homeostasis, Feed efficiency traits, GWAS, Regulatory pathways

## Abstract

**Background:**

Feed efficiency (FE) related traits play a key role in the economy and sustainability of beef cattle production systems. The accurate knowledge of the physiologic background for FE-related traits can help the development of more efficient selection strategies for them. Hence, multi-trait weighted GWAS (MTwGWAS) and meta-analyze were used to find genomic regions associated with average daily gain (ADG), dry matter intake (DMI), feed conversion ratio (FCR), feed efficiency (FE), and residual feed intake (RFI). The FE-related traits and genomic information belong to two breeding programs that perform the FE test at different ages: post-weaning (1,024 animals IZ population) and post-yearling (918 animals for the QLT population).

**Results:**

The meta-analyze MTwGWAS identified 14 genomic regions (-log10(*p *-value) > 5) regions mapped on BTA 1, 2, 3, 4, 7, 8, 11, 14, 15, 18, 21, and 29. These regions explained a large proportion of the total genetic variance for FE-related traits across-population ranging from 20% (FCR) to 36% (DMI) in the *IZ* population and from 22% (RFI) to 28% (ADG) in the *QLT* population. Relevant candidate genes within these regions *(LIPE, LPL, IGF1R, IGF1, IGFBP5, IGF2, INS, INSR, LEPR, LEPROT, POMC, NPY, AGRP, TGFB1, GHSR, JAK1, LYN, MOS, PLAG1, CHCD7, LCAT*, and *PLA2G15*) highlighted that the physiological mechanisms related to neuropeptides and the metabolic signals controlling the body's energy balance are responsible for leading to greater feed efficiency. Integrated meta-analysis results and functional pathway enrichment analysis highlighted the major effect of biological functions linked to energy, lipid metabolism, and hormone signaling that mediates the effects of peptide signals in the hypothalamus and whole-body energy homeostasis affecting the genetic control of FE-related traits in Nellore cattle.

**Conclusions:**

Genes and pathways associated with common signals for feed efficiency-related traits provide better knowledge about regions with biological relevance in physiological mechanisms associated with differences in energy metabolism and hypothalamus signaling. These pleiotropic regions would support the selection for feed efficiency-related traits, incorporating and pondering causal variations assigning prior weights in genomic selection approaches.

**Supplementary Information:**

The online version contains supplementary material available at 10.1186/s12864-022-08671-w.

## Background

The genetic improvement of animal feed efficiency (FE) has been considered a relevant aspect in breeding programs to achieve reductions in feed costs and environmental impacts [1]. Indeed, feeding can make up to 70% of the total beef cattle production costs [2]. Therefore, selecting animals for FE-related traits improve the profitability of beef cattle production systems by increasing productivity while reducing feed intake [3]. Usually, the FE in beef cattle is evaluated by dry matter intake (DMI), feed conversion ratio (FCR), feed efficiency (FE), and residual feed intake (RFI). However, the accurate measurement of FE-related traits is expensive and difficult, limiting the number of animals evaluated.

Feed efficiency-related traits are controlled by a complex interaction of different physiological mechanisms and biological processes regulating feed intake and energy expenditure [4–6]. Genome-wide association studies (GWAS) have been pointed out major genomic regions involved in physiological factors responsible for the phenotypic variation in FE-related traits in beef cattle populations [7–10]. However, FE-related traits are controlled by several quantitative trait loci (QTL) with small effects, and applying GWAS can generate false-positive marker signals in small populations, requiring a large population to accurately detect the SNP markers with small or moderate additive effect [11].

Assembling a large enough population for accurate detection of QTLs is the major challenge for GWAS, especially for FE-related traits which are commonly evaluated in small populations. A limited number of animals with genotype and phenotypic information lead to lower accuracy of SNP markers effect, making it difficult to identify causative mutations directly associated with the target trait [11]. In this context, meta-analysis is an efficient approach to overcome this limitation related to population size by combining results from independent studies in order to increase the power of detection and map genomic variants accurately affecting the trait and decrease false-positive associations [12–14]. In addition, using GWAS meta-analysis to identify genomic regions can provide better knowledge of the main biological mechanisms involved in genetic architecture regulation of FE-related traits in different populations of Nellore cattle[9, 10]. Thus, the knowledge regarding genomic regions and biological pathways involved with differences in FE-related traits across Nellore populations could aid the development of more efficient strategies and tools to attain genetic improvement of more efficient animals. Hence, this study was carried out to uncover potential genomic regions and candidate genes acting in biological functions for FE-related traits across two Nellore breeding populations.

## Material and methods

The FE-related traits (ADG, DMI, FCR, FE, and RFI) and genomic information were obtained for 1,024 animals belonging to an experimental breeding program at Beef Cattle Research Center (Institute of Animal Science – IZ), and 918 animals from a commercial breeding program Nellore Qualitas (QLT). The animal procedures realized in this research agreed with Animal Care of the São Paulo State University (UNESP), School of Agricultural and Veterinary Science Ethical Committee (protocol number 18.340/16).

### Experimental breeding program – IZ population

The experimental breeding program was established in 1980, selecting animals based on yearling body weight (YBW) measured at 378 days of age in young bulls and 550 days of age in heifers. The *IZ* population is divided into three selection herds: Nellore control (NeC), Nellore Selection (NeS), and Nellore Traditional (NeT). In NeC, animals are selected based on differential selection for YBW close to zero. On the other hand, animals are selected for the maximum differential selection for YBW in NeS and NeT [15]. In the NeT, sires from commercial herds and sires from the NeS can be used in the breeding season. The NeC and NeS are closed herds, i.e., only sires from the same herd are used in the breeding season, and the inbreeding rate is controlled in all the herds with planned mattings.

The FE-related traits were measured on 1,156 animals (801 males and 355 females), born from 2004 to 2015, being 146 of NeC (104 males and 42 females), 300 of NeS (214 males and 86 females), and 710 of NeT (483 males and 227 females) herd. The animals were evaluated in a feeding trial, in which they were either housed in individual pens (683 animals) or group pens equipped with the GrowSafe feeding system (473 animals). In both situations, the feeding trial comprised at least 21 days for adaptation to the feedlot diet and management and at least 56 days for the data collection period. During the feeding trial period, the animals were grouped according to sex, males exhibited an average age at the beginning of the trial of 275 and 366 ± 27.5 days in the end, while females showed an average age at the beginning of the trial of 302 and 384 ± 45.4 days at the end of the feeding trial.

Animals were weighed without fasting at the beginning and the end of the feeding trial, as well as every 14 days during the experimental period. The mixed diet (dry corn grain, corn silage, soybean, urea, and mineral salt) was offered *ad libitum* and formulated with 67% of total digestible nutrients (TDN) and 13% of crude protein (CP), allowing an average daily gain (ADG) of 1.1 kg/day.

### Commercial breeding program – Nelore Qualitas population

Nelore Qualitas (*QLT*) breeding program comprises 34 farms distributed in two regions of Brazil (Midwest and Southeast) and Bolivia. The animals are selected based on a selection index that includes body weight at weaning (24%), weight gain from weaning to yearling (38%), scrotal circumference (19%), and muscling visual score (19%), both measured around 15 months of age. In the *QLT* population, FE-related traits from 947 young bulls, born from 2008 to 2015, were evaluated in feeding trials in individual pens (715 animals) or group pens equipped with the Intergado feeding system (232 animals). The Intergado System operates like the GrowSafe System, measuring the animals' feed intake and feed frequency. Each feeding trial comprised 28 days for adaptation to the feedlot diet and 56 days for data recording. Animals were weighed without fasting, at the beginning and ending, as well as every 21 days during the experimental period. The diet (dry corn grain, corn silage, sugarcane bagasse, soybean, urea, mineral salt, and potassium chloride) was offered ad libitum and formulated with 82% of total digestible nutrients (TDN) and 12,5% of CP, allowing an ADG of 1.5 kg/day. Animals were, on average, 652 ± 38.43 days old at the beginning and 712.02 ± 38.43 days old at the end of the feeding trial.

### Phenotypic traits

During the feeding trials, in both *IZ* and *QLT* populations, the mixed diet was offered at 8:00 h and 16:00 h, allowing from 5 to 10% refusals. In the individual pens, the orts were weighed daily in the morning before the feed delivery to calculate the daily dietary intake. In the group pens, the feed intake was recorded automatically by the GrowSafe or Intergado feeding system. Thus, the DMI was estimated as the feed intake by each animal with subsequent adjustment for dry matter content and expressed as kg/day. Average daily gain (ADG) was defined as the slope from the linear regression of body weight (BW) on feeding trial days. The ratio between ADG and DMI was used to calculate the feed efficiency (FE), and the ratio between DMI and ADG was used to estimate the feed conversion rate (FCR). Residual feed intake (RFI) was calculated in each test year as the difference between the observed and expected feed intake considering the each animal's average metabolic body weight (MBW) and ADG, using the gold-standard equation proposed by Koch et al. [16] as follows:

$$DMI= {\beta }_{0}+{\beta }_{1}ADG+{\beta }_{2}{MBW}^{0.75}+\varepsilon$$

where ${\beta }_{0}$ is the intercept, ${\beta }_{1}$ and ${\beta }_{2}$ are the linear regression coefficients for $ADG$ and ${MBW}^{0.75}$, respectively, and $\varepsilon$ is the residue of the equation that represents the RFI estimate.

Phenotypic data quality control was performed for each breeding program (*IZ* and *Qualitas*), excluding records outside the interval of ± 3.5 *SD* below or above the mean of each contemporary group (CG). After phenotypic quality control, the number of animals per CG ranged from 10 to 70 for the *IZ* population and from 27 to 119 for the *QLT* population. The CG in the *IZ* population was defined by sex and month and year of birth and in the *QLT* population by month and year of birth and year of feed trial. In both cases, for animals in the group pens, the feed trial pen was added. Descriptive statistics and genetic parameter estimate for FE-related traits are reported in Table 1.

**Table 1:** Descriptive statistics, additive genetic variance (${\sigma }_{a}^{2}$) and heritability (${h}^{2}$) estimates for Nellore feed efficiency-related traits in *IZ* and *Qualitas populations*

*IZ population (N* = *1,024)*						
Trait	Mean	SD	Min	Max	${\sigma }_{a}^{2}$	${h}^{2}$
ADG (kg/day)	1.04	0.25	0.17	1.72	0.010 (0.002)	0.33 (0.042)
FCR	7.24	1.66	3.65	15.23	0.19 (0.072)	0.19 (0.034)
RFI (kg/day)	-0.01	0.61	-2.36	4.13	0.27 (0.052)	0.28 (0.022)
DMI (kg/day)	7.29	1.54	2.15	12.64	0.32 (0.033)	0.40 (0.039)
FE	0.15	0.03	0.07	0.27	0.001 (0.0004)	0.21 (0.029)
*Qualitas population (N* = *918)*						
ADG (kg/day)	1.53	0.43	0.28	2.48	0.028 (0.007)	0.34 (0.018)
FCR	7.88	3.05	4.32	21.79	1.13 (0.302)	0.23 (0.017)
RFI (kg/day)	-0.006	0.76	-2.78	3.34	0.35 (0.035)	0.35 (0.025)
DMI (kg/day)	11.05	1.44	7.18	15.84	0.47 (0.092)	0.45 (0.019)
FE	0.13	0.03	0.05	0.23	0.008 (0.0005)	0.30 (0.022)

*ADG* average daily gain, *FCR* feed conversion ratio, *FE* feed efficiency, *RFI* residual feed intake, *DMI* dry matter intake and ${h}^{2}$ – in parentheses represents a standard error for heritability estimates

### Genotype dataset

A total of 846 (*IZ*) and 582 (*QLT*) animals were genotyped using the Illumina BovineHD BeadChip assay (770 k, Illumina Inc., San Diego, CA, USA) and 310 (*IZ*) and 372 (*QLT*) animals using GeneSeek® HDi 75 K (GeneSeek In/c., Lincoln, NE). For each population, animals genotyped with the lower density panel were imputed to the HD panel using FImpute v2.2 [17] separately, and its expected accuracy was higher than 0.97. In the genotype quality control, we removed non-autosomal markers and those presenting minor allele frequency (MAF) less than 0.03, significant deviating from Hardy–Weinberg equilibrium (P $\le$ 10^–5^), and with call rate less than 0.90. In addition, samples with a call rate lower than 0.90 were also removed from the analyses. After quality control, 1,024 animals from *IZ* and 918 animals from *QLT,* and 387,035 SNP markers in common between the two populations remained in the dataset.

Principal component analysis (PCA) evaluated the population substructure based on the SNP markers using the ade4 R package [18]. Four groups have been clustered using k-means clustering, three groups from the IZ population and one from the *QLT* population (Supplementary Fig. S1). The animals' dispersion in the PCA plot indicated the absence of subgroups in the *QLT* population.

### Genome-wide association study

Multi-trait weighted genome-wide association analysis (MTwGWAS) was performed for each population, separately, considering the following general model:

$$y=X\beta +Za+e$$

where $y$ is the matrix of FE-related traits; $\beta$ is the vector of fixed effects; $a$ is the additive effect of animals and $e$ is the residual effect. The $X$ and $Z$ correspond to the incidence matrices related to fixed and random effects, respectively. Fixed effects were: CG, age at the beginning of the feed trial as linear and quadratic co-variables for both populations, and the *IZ* population*,* the three first principal components that explain 7.55% of the genotypic variability and the linear and quadratic effect of cow age were also considered.

The random effects of animal and residual were assumed to be normally distributed: $a\sim (0,G\otimes {S}_{a}$) and $e\sim (0,I\otimes$ R), where $G$ is the genomic relationship matrix according to VanRaden [19], ⊗ is the Kronecker product, $I$ is the identity matrix, ${S}_{a}$ is a (co)variance matrix of direct additive and $R$ is a (co)variance matrix of residual effects for FE related traits, respectively. The ***G*** matrix used in the MTwGWAS method was constructed as follows: $G=MD{M}^{^{\prime}}q$ where $M$ is the SNP matrix assuming 0, 1, and 2 for genotypes AA, AB, and BB; $D$ is a diagonal weight matrix for each SNP marker and $q$ is a weighting factor given as $q=\frac{1}{{\sum }_{j=1}^{m}2{p}_{j}\left(1-{p}_{j}\right)}$ where ${p}_{j}$ is the second allele frequency of the *jth* SNP marker.

The SNP marker effect and weights were calculated based on genomic breeding values (GEBV) of genotyped animals obtained from a multi-trait model through the algorithm proposed by Wang et al. [20]. The postgs_trt_eff option of the POSTGSF90 program [21] allows considering the MTwGWAS analysis specifying the trait for which the SNP marker effect will be estimated each trait per time. The MTwGWAS is an iterative approach, but considering many iterations to calculate SNP weights can cause subjective peaks [20]. Therefore, to maximize the accuracy of SNP marker signals detection, the analysis was run using two iterations to estimate the genetic variance explained by the markers [22].

The SNP marker effect and weights for wGWAS were estimated considering the algorithm proposed by Wang [20]: 1) In the first step *D* = ***I*** and second step *D* = *w* (*w* were the weight estimates obtained in step 6); 2) Calculate the G matrix ($G=MD{M}^{^{\prime}}q$); 3) Estimation of the GEBV for animals using the multi-trait GBLUP; 4) Estimation of the SNP marker effect ($\widehat{u}$) based on the GEBV ($\widehat{a}$) of animals from the equation $\widehat u=DM'\left[MDM'\right]^{-1}{\widehat{a}}$; 5) Estimation of the SNP marker weight ($D$) as follows: $D={\widehat{u}}^{2}2{p}_{j}\left(1-{p}_{j}\right)$ where ${\widehat{u}}^{2}$ is the allele substitution effect of each SNP marker and 6) the SNP markers weight (D) are normalized to keep the total genetic variance constant, to use in step 2.

Results from MTwGWAS was used to estimate the proportion of genetic variance explained by SNPs markers (${\sigma }_{\widehat{u}}^{2}$) as follows: ${\sigma }_{\widehat{u}}^{2}=\frac{Var({\sum }_{j=1}^{100}{Z}_{j}{\widehat{u}}_{j})}{{\sigma }_{a}^{2}} x 100 \%$, where ${\sigma }_{a}^{2}$ is the genetic variance for each FE-related trait; ${Z}_{j}$ is the vector of *jth* SNP marker and ${\widehat{u}}_{j}$ is the SNP effect of the *jth* SNP within the window with 100 markers.

### Detection of candidate genomic regions across the population

To identify the regions affecting FE-related traits from GWAS analyzes performed separately for each population (IZ and QLT), a multi-trait meta-analysis statistical method, described by Bolormaa et al. [14], was performed. For statistical tests, the SNP effects were standardized as follows: ${t}_{k}=\frac{{\widehat{u}}_{k}}{SE({\widehat{u}}_{k})}$, where, ${t}_{k}$ is the *t *-values for the SNP marker effect; ${\widehat{u}}_{k}$ is the SNP effects for each trait in each population and $SE({\widehat{u}}_{k})$ is the standard error for SNP effect (${\widehat{u}}_{k}$).

A multi-trait meta-analysis statistic test was used to evaluate the association and influence of SNP effects for FE-related traits across two Nellore cattle populations [14]. This statistic test summarizes single-marker statistics following a ${\chi }^{2}$ distribution with *k* degrees of freedom, where *k* is the number of traits included in the multi-trait statistical test. For each SNP marker (total of 387,035 SNP markers) the statistic was: $Multi-trait {\chi }^{2}={t}_{k}^{^{\prime}}{V}^{-1}{t}_{k}$, where ${t}_{k}$ is a vector 10 × 1 of the signed *t* -value of SNP_k_ for the 5 FE-related traits in the 2 Nellore cattle population, ${t}_{k}^{^{\prime}}$ is a transpose of vector ${t}_{k}$; ${V}^{-1}$ is an inverse of the *t *-values correlation matrix between the *t *-values (10 × 10). The ${V}^{-1}$ was corrected by adding the average correlation of each trait to its respective diagonal element [23]. This correction was used because some traits show higher correlations than others and may lead to highly significant composite scores even when single-trait analyses have lower evidence of the association [23]. The *p *-value adjustment for multiple tests was performed using the false discovery rate (FDR) test [24]: $fdr=\frac{{m}_{SNP}}{{s}_{SNP}}*\alpha$, where ${m}_{SNP}$ represent the number of SNP markers considered in the analyze (387,035), $\alpha$ is the significance threshold (*p *-value < 0.05) and ${s}_{SNP}$ is related to the number of significant markers with *p *-value < $\alpha$.

### Gene mapping and functional gene enrichment analysis

The SNP markers from statistical combination were deemed significant when –log10(*p *-value) > 5.0 (5% FDR) and grouped in SN*P*-window regions within each BTA when the markers did not show a gap greater than 0.2 Mb among them. Genes in significant SN*P*-window regions were identified using the NCBI BioSystems database for cattle using the map *Bos taurus* ARS-UCD1.2 assembly as reference. The candidate gene list from meta-analysis GWAS was used as the target gene list for functional classification for biological process (BP; Gene Ontology—GO) and KEGG pathways using the string R Package [25] considering the Bovine database background [26]. The functional annotation was considered significant at a *p *-value < 0.05 and an FDR of 5% for multiple test correction as described by Boyle et al. [27].

## Results and discussion

### Significant genomic regions

**Fig. 1:**
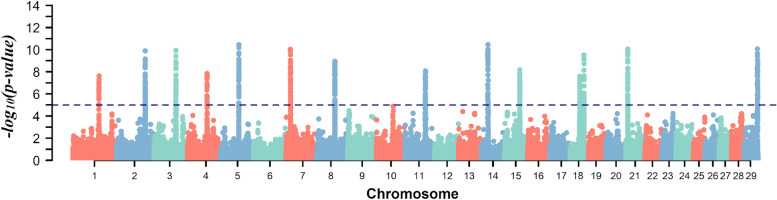
Manhattan plot for the statistical combination of genome-wide results for feed efficiency-related traits, average daily gain (ADG), dry matter intake (DMI), feed conversion ratio (FCR), feed efficiency (FE), and residual feed intake (RFI) across Nellore cattle populations. The horizontal blue line represents the significance threshold -log10(*p *-value) > 5.0 for markers considering an FDR of 5%

Multi-trait meta-analysis of the FE-related traits across Nellore cattle populations identified a total of fourteen genomic regions ($-{log}_{10}(p-value)$ > 5), mapped on BTA 1, 2, 3, 4, 7, 8, 11, 14, 15, 18, 21, and 29 (Fig. 1). These regions explained a large proportion of the genetic variance for FE-related traits that IZ (26.99% for ADG, 30.83% for DMI, 17.69% for FCR, 34.04% for FE and 33.76% for RFI) and QLT (27.82% for ADG, 26.85% for DMI, 22.34% for FCR, 22.86% for FE and 24.40% for RFI) Nellore populations shared. Most significant SNP markers were mapped on intron and downstream of gene variants (81.93%), capturing a relatively greater proportion of the additive genetic variance than other functional classes. Although in lower proportion, SNP markers mapped in missense (3.65%) and splicing (0.65%), 3' (0.73%), and 5' (0.54%) prime UTR variants, these regions play a key role in gene expression regulation and translation [28, 29].

The SN*P*-window on BTA1 (94.55 – 95.90 Mb) explained more than 2% of the genetic variance for FE-related traits (Supplementary Fig. 2, 3 and 4). This region harbors the major genes *NCEH1, GHSR, ECT2*, *GPX6,* and *GPX5* affecting physiological processes with important effects in the regulation of pituitary growth hormone secretion, feed intake, and energy homeostasis (Table 2). The *NCEH1* gene has been associated with lipid metabolism and was under-expressed in the muscle of pigs phenotypically classified for high fatty acid composition [30]. The *GHSR* gene shows a striking effect in regulating energetic homeostasis, insulin sensitivity and glucose uptake, and ghrelin secretion, with a major role in body weight and growth efficiency [31]. The *GPX6* and *GPX5* genes are involved in adaptive responses to oxidative stress by their antioxidant action, resulting in great tolerance to oxidative stress in animals with high feed efficiency [32]. Thus, these genes negatively control the actions of mitochondrial reactive oxygen species (ROS) on metabolism, representing an important physiological mechanism in more efficient animals [32].

**Table 2:** Genes surrounding the significant genomic regions (-*log*_*10*_*(p *-value*)* > *5*) identified using the multi-trait meta-analysis statistical test of genome-wide association (GWAS) results for Feed efficiency-related traits (FE, FCR, RFI, and its components ADG, DMI), in *IZ* and Qualitas populations

BTA	Windows (Mb)^a^	*IZ* population Traits^b^	Qualitas population Traits^b^	Genes
1	94.55—95.90	ADG, DMI, FE, RFI	ADG, DMI, FCR, RFI	ECT2, NCEH1, TNFSF10, GHSR, FNDC3B, TMEM212, PLD1, STXBP5L, POLQ, SPATA16
2	104.14—105.06	ADG, DMI, FCR, FE, RFI	ADG, DMI, FCR, FE, RFI	XRCC5, MARCH4, SMARCAL1, RPL37A, IGFBP2, IGFBP5, TRNAS-GGA, TNP1
3	78.99—80.84	DMI, FE	ADG, DMI	PDE4B, MGC137454, LEPR, LEPROT, DNAJC6, AK4, JAK1, bta-mir-101–1, RAVER2, CACHD1
4	70.88—71.85	ADG, DMI, FCR, FE, RFI	FCR, FE	OSBPL3, GSDME, MPP6, NPY
5	65.95—67.03	FCR, RFI	FE	NUP37, PARPBP, PMCH, IGF1, PAH, ASCL1, U1
7	15.60—16.54	ADG, DMI, FCR, FE, RFI	ADG, FCR, FE	KANK2, ACP5, ANGPTL8, ARHGEF18, CAMSAP3, CCDC151, CCDC159, CNN1, DOCK6, ECSIT, ELAVL3, ELOF1, EPOR, FCER2, INSR, MBD3L3, MCOLN1, PCP2, PET100, PEX11G, PLPPR2, PNPLA6, PRKCSH, RAB3D, RETN, RGL3, STXBP2, SWSAP1, TEX45, TMEM205, TRAPPC5, TSPAN16, U4, VN2R404P, XAB2, ZNF358, ZNF557, ZNF653
8	66.71—67.82	ADG	DMI, RFI	LPL, SLC18A1, ATP6V1B2, LZTS1
11	73.89—74.76	ADG, DMI, FCR, FE, RFI	DMI, FCR, RFI	DTNB, DNMT3A, bta-mir-1301, POMC, EFR3B, DNAJC27, ADCY3, CENPO, PTRHD1, NCOA1
14	22.62—24.71	ADG, DMI, FCR, FE, RFI	ADG, DMI, FCR, FE, RFI	FAM110B, LYN, XKR4, TMEM68, RPS20, TMEM68, TGS1, TRNAT-AGU, U1, LYN, MOS, PLAG1, CHCHD7, SDR16C5, SDR16C6, PENK, U6, IMPAD1, FAM110B, UBXN2B, CYP7A1
15	56.22—56.63	ADG	ADG	ACER3, B3GNT6, CAPN5, OMP, MYO7A
18	35.00—35.79	FE	DMI, RFI	CTCF, CARMIL2, ACD, PARD6A, ENKD1, GFOD2, RANBP10, TSNAXIP1, CENPT, THAP11, NUTF2, EDC4, NRN1L, PSKH1, PSMB10, LCAT, SLC12A4, DPEP3, DPEP2, DDX28, DUS2, NFATC3, ESRP2, PLA2G15, SLC7A6, SLC7A6OS, PRMT7, SMPD3, TPPP3, ZDHHC1, HSD11B2, ATP6V0D1, AGRP, RIPOR1, CTCF, C18H16orf86
18	49.66—50.93	DMI, RFI	ADG, FCR, FE, RFI	ITPKC, SNRPA, MIA, RAB4B, bta-mir-12057, EGLN2, CYP2F1, CYP2B6, CYP2S1, AXL, HNRNPUL1, TGFB1, B9D2, TMEM91, EXOSC5, BCKDHA, B3GNT8, DMAC2, ERICH4, CEACAM1, LIPE, PLD3, HIPK4, PRX, SERTAD1, SERTAD3, BLVRB, SPTBN4, SHKBP1, LTBP4, NUMBL, COQ8B, CCDC97, C18H19orf47, C18H19orf54
21	7.62—8.15	ADG, DMI, FE, RFI	DMI, RFI	IGF1R, PGPEP1L
29	48.75—50.42	FCR, RFI	ADG, FCR, FE	KCNQ1, TRPM5, TSSC4, CD81, TSPAN32, ASCL2, TH, INS, IGF2, MRPL23, TNNT3, LSP1, TNNI2, SYT8, CTSD, IFITM10, DUSP8, MOB2, TOLLIP, AP2A2

^a^ The windows region represents the lower and maximum position for SNP markers deemed significantly ($-{log}_{10}(p-value)$ > 5) from the multi-trait statistic test combination

^b^ Trait in which the genomic regions was significant using a multi-trait weighted genome-wide association studies (GWAS)

The SN*P*-window on BTA2 (104.14 – 105.06 Mb) explained more than 2.80% of additive genetic variance (Supplementary Fig. 2, 3 and 4). This region is surrounded by the major genes *IGFBP2* and *IGFBP5* (Table 2), which are associated with IGF-mediated functions directly related to variations on energy expenditure in muscle and physiological mechanisms contributing to improving feed efficiency in cattle [33, 34]. The region on BTA14 (22.62 – 24.71 Mb; Table 2) explained more than 1.40% of additive genetic variance and harbors the genes *LYN*, *TMEM68*, *PLAG1*, *CHCHD7*, *MOS*, *PENK,* and *IMPAD1,* which affect mostly MAPK signaling pathway, mechanisms related to cell proliferation and growth factors such as *IGF* 1 and 2 with an effect on feeding control by energy metabolism and linked to tissue development [35, 36]. Studies have indicated the BTA14 as a functional pleiotropic region underlying genetic differences in residual feed intake and its component traits DMI, ADG, and metabolic body weight [37]. The region on BTA 29 (48.78 – 50.42 Mb) explained from 2.14% to 4.97% of the genetic variance (Supplementary Fig. 2, 3 and 4)*.* This region harbors genes such as *INS*, *IGF2,* and *TH* (Table 2) with a major effect on animal metabolism related to insulin and glucose signaling pathways, controlling the energy homeostasis by their effect on fatty acids, glycerol, glucose, and acetyl CoA [23, 38]. The action of genes related to energy homeostasis results in differences in the mechanisms of feed intake due to energy homeostasis and growth[39],. Besides, this region surrounds genes involved in the energy metabolism of skeletal muscle (TNNT3 and TNNI2). Such findings support the hypothesis that changes in energy expenditure by muscle contraction are associated with differences in FE [40, 41]. Thus, animals with a lower energy requirement for the maintenance of skeletal muscle tissue show greater feed efficiency [37].

The genes identified on BTA3 (78.99 – 80.84 Mb), BTA4 (70.88 – 71.85 Mb), and BTA11 (73.89 – 74.76 Mb; Fig. 1), explained a substantial amount of genetic variance, ranging from 1.19% to 4.95%, and the regions show a key role in neuroendocrinal signal affecting feed intake (Supplementary Fig. S2, S3 and S4). These regions harbor functional genes that play a role in the peptide signal-regulating feed intake and energy expenditure as *LEPR, LEPROT,* and *JAK1* on BTA3, *OSBPL3,* and *NPY* on BTA4, and *POMC* and *ASXL2* on BTA11 (Table 2). These genes play a specific hypothalamic function and are metabolic modulators that strongly contribute to differences in feed intake and energy homeostasis [42]. The genes *LEPR, LEPROT,* and *JAK1,* are directly associated with feed intake by their major role in physiological body homeostasis and association with the genes *Leptin* and *NPY*[4, 43]. Mota et al. [4] observed that serum leptin levels and their gene expression probably control the feed intake in young Nellore bulls, and Karisa et al. [43] reported that these genes are associated with biological processes leading to more efficient animals. The *OSBPL3* gene acts as a lipid transporter or sensor at membrane contact sites, affecting lipid metabolism [44]. Changes in physiological mechanisms related to lipid metabolism have been indicated to affect the feed efficiency in beef cattle [6]. The *NPY* gene plays a functional connection in the major physiological mechanism regulating feed intake, growth, and energy, and its action represents an important factor affecting FE-related traits [45, 46]. The *POMC* gene, on BTA11, is an appetite-related neuropeptide associated with the neuronal control of key mechanisms by which animals regulate the feeding intake and body energy homeostasis in cattle [47], sheep [45], and chicken [42]. The gene *ASXL2* plays an important role in adipogenesis and acts as a coactivator for proliferator-activated receptor gamma (*PPARG*) associated with feeding control [48]. The SN*P*-window regions on BTA 3, 4, and 11 might directly affect FE-related traits due to the potential regulation of energy metabolism and neuroendocrine pathways, with major effects on catabolic and anabolic pathways involved in feed intake control and energy homeostasis.

The major candidate genes identified on BTA5 (65.95—67.03 Mb) were: *IGF1, PMCH,* and *PARPBP* (Table 2)*.* The *IGF1* gene has functions on insulin metabolism, muscle adaptation, and average daily gain, whereas the *PMCH* gene is related to carcass fat levels and marbling score [49]. The *PARPBP* gene regulates the activity of the *PARP1* gene, which plays an important role in the cell cycle and metabolism through insulin resistance [50]. The genes identified on BTA7 showed an important effect on muscle metabolism (*ARHGEF18* and *CNN1*), lipid regulation (*ANGPTL8*), and energy metabolism (*EPOR, INSR,* and *RETN*; Table 2)*.* The genes *ARHGEF18* and *CNN1* affect the energy expenditure in the skeletal muscle required for maintenance, contributing to increasing feed efficiency by reducing oxidative stress (*ARHGEF18)* and affecting the oxidative metabolism in muscle fibers (*CNN1*) [51]. The gene *ANGPTL8* plays functions in physiological adaptation through lipid and glucose homeostasis, affecting bovine's adipogenesis [52]. These factors have been associated with energy balance through their impact on the concentration of circulating metabolites (insulin and glucose), one of the main metabolic factors for increasing efficiency in Nellore cattle [53]. Reyer et al. [54] observed that pigs with high FE showed less hepatic fat content than low FE, reflecting reductions in uptake/storage of fatty acids. The genes affecting energy metabolism occur by the mediation of JAK2/STAT5 (*EPOR)*, energy balance by specific anabolic and catabolic pathways (*INSR*), as well as in glucose and lipid metabolism by the action of the resistin gene (*RETN*). The genes *EPOR, INSR,* and *RETN* could be involved as peripheral signals of energy homeostasis controlled by glucose and insulin homeostasis leading to important feedback with *NPY* regulating the feed intake [55].

The *LPL* gene mapped on BTA8 (65.95 – 67.82 Mb) is directly involved in the metabolism and transport of lipids. Montanholi et al. [56] and Karisa et al. [43] suggested that less lipogenesis, lipid transport, and fat deposition occurs in beef cattle with high feed efficiency. The LPL gene is a multifunctional enzyme involved in energy requirements in cattle, affecting feed intake, glucose, and lipids metabolism [57]. The genes *CAPN5* and *MYO7A* identified on BTA15 (56.22 – 56.63 Mb) are associated with muscle metabolism (Table 2). The *CAPN5* regulates the rate of cells' proteolytic changes and can control cell growth, differentiation, and apoptosis [58, 59]. The *MYO7A* is related to the myosin family with a moderate effect on DMI in beef cattle [9]. In mice, homozygous for this region displayed decreased body weight and fat [60]. Thus, these genes might be directly associated with enhanced growth efficiency by regulating fat and muscle deposition ratio [59].

The genomic region mapped on BTA18 (35.00 – 35.79 Mb) accounted from 3.30% to 4.95% of genetic variance for FE-related traits (Supplementary information Fig. S3 and S4). A total of 36 genes surrounding this region was found from these; the gene set, including *LCAT, PLA2G15, ATP6V0D1, AGRP,* and *RIPOR1*, is involved with potential mechanisms related to feeding intake control. Down-regulation of the gene *LCAT* decreases the HDL (High-Density Lipoproteins) formation leading to a reduction in the capacity to transport cholesterol from adipose tissue to liver and muscle. The *PLA2G15* gene regulates the hydrolysis of phospholipids into free fatty acids. There is evidence that high feed efficient animals exhibit reduced hepatic usage of fatty acids [61]. The gene *AGRP* is associated with hypothalamic integration of energy balance, nutrient partition control, and feed intake increase by antagonizing the effects of the orexigenic peptides [42].

The genomic region on BTA18 (49.66 – 50.93 Mb) explained a significant amount of genetic variance from 2.05% to 4.07% (Supplementary information Fig. S2, S3 and S4). The major genes identified in this window were *LTBP4, TGFB1, CYP2F1, CYP2B6, CYP2S1,* and *LIPE.* The *LTBP4* gene is a key regulator of *TGFB* (transforming growth factor-beta) and *TGFB1.* It is associated with skeletal muscle development and growth, a main biological factor affecting feed-related traits. Jing et al. [62] in pig and Alexandre et al. [5] in Nellore cattle observed that the *TGFB1* signaling pathway plays a key effect in feed efficiency by skeletal muscle growth stimulation and metabolism. The gene set (*CYP2F1, CYP2B6,* and *CYP2S1)* is a member of cytochrome P450 proteins involved in synthesizing steroids and lipids. Tizioto et al. [6] observed that the cytochrome P450 family was down-regulated in Nellore cattle less efficient, indicating high oxidative stress in these animals. The *LIPE* gene codifies an enzyme with function in lipid hydrolysis, mainly hormone-sensitive lipase (*HSL*). The LIPE gene increases lipolysis during the negative energy balance in dairy cattle to attend to energy homeostasis [63]. Thus, animals with the greatest FE might show a higher tolerance for oxidative stress leading to lower energy expenditure and greater tissue metabolism.

A genomic region on BTA21 explained from 2.47% to 4.56% of the genetic variance (Supplementary Fig. S2, S3 and S4). This genomic region surrounds the *IGF1R* gene, which substantially affects genetic differences in body weight and feed efficiency in cattle [39, 64]. Kelly et al. [39] observed an over-expression of the *IGF1R* gene associated with energy efficiency in more efficient animals. Abo-Ismail et al. [64] observed a significant effect of this gene on ADG and marbling. In this context, the potential effect of the *IGF1R* gene on FE-related traits is through changes in target metabolic pathways affecting the energy balance, protein synthesis, or breakdown.

### Functional enrichment of potential candidate genes

Enrichment analysis results pointed out pathways that link whole-body energy balance through neuropeptides, hormones, and metabolites to maintain the greatest feed efficiency (Table 3). In addition, these results obtained from biological process (BP) and KEGG pathways enrichment analyses highlighted that FE-related traits share common biological pathways and underlie the relevance of pleiotropy in important physiological events that regulate cattle feed efficiency (Supplementary Table S1 and S2).

**Table 3:** Gene enrichment analysis for enriched KEGG pathways, related to gene set identified using the multi-trait meta-analysis statistical test for feed efficiency related traits

Pathway ID	Description	*p *-value	q -value	Gene ID
bta04923	Regulation of lipolysis in adipocytes	0.00004	0.00470	LIPE, ADCY3, INSR, INS, NPY
bta04152	AMPK signaling pathway	0.00017	0.00630	LIPE, IGF1R, IGF1, INSR, LEPR, INS
bta04015	Rap1 signaling pathway	0.00045	0.01040	PARD6A, ADCY3, IGF1R, IGF1, INSR, INS
bta04010	MAPK signaling pathway	0.00080	0.01040	TGFB1, IGF1R, ECSIT, IGF1, INSR, INS, IGF2
bta04014	Ras signaling pathway	0.00088	0.01040	IGF1R, IGF1, INSR, PLD1, INS, IGF2
bta04068	FoxO signaling pathway	0.00190	0.01770	TGFB1, IGF1R, IGF1, INSR, INS
bta04024	cAMP signaling pathway	0.00200	0.01780	GHSR, LIPE, ADCY3, PDE4B, PLD1, NPY
bta04910	Insulin signaling pathway	0.01000	0.04380	INS, INSR, LIPE
bta04935	Growth hormone synthesis, secretion, and action	0.02690	0.04140	ADCY3, GHSR, IGF1
bta04630	JAK-STAT signaling pathway	0.03470	0.04710	EPOR, JAK1, LEPR

### KEGG pathway

Enriched pathways identified the MAPK (bta04010), AMPK (bta04152), and insulin signaling (bta04910) pathways which play a key role in molecular signals that act on energy homeostasis leading to the greatest feed efficiency in cattle (Table 3). The MAPK (bta04010) and insulin signaling (bta04910) pathways have a crucial role in the regulatory pathways of the biological responses to the insulin and IGF-1 levels, leading to differences in energy metabolism through glucose and lipid metabolism [65]. The control energy metabolism by u*p*-regulating genes associated with MAPK signaling could explain their relationship with animals selected to increase feed efficiency [66].

The AMPK pathway (bta04152) is associated with metabolic energy balance 67 and increases protein metabolism, fatty acid oxidation, glucose uptake, and glycolysis (Table 3). Thus, the AMPK pathway regulates these principal energy sources, maintaining energy balance at the whole-body level by mediating effects on different hormones acting on hypothalamic regions, which regulate feed intake and energy expenditure [67, 68]. In this context, Hu et al. [69] observed a striking effect of AMPK on bird feed intake control through regulation of nutritional status and energy homeostasis. Thus, the feed intake control mediated by pathways related to AMPK and the neuroactive receptor-ligand interaction (bta04080) were related to genomic regions that harbor genes with actions in the arc from the nucleus of the hypothalamus (*NPY, POMC, PENK, GHSR, LEPR*, and *LEPROT*) [68]. This result increased the evidence of genomic regions mediating appetite modulation and metabolic effects in controlling feed intake in populations (IZ and QLT).

The significant metabolic pathways JAK-STAT signaling (bta04630), insulin signaling (bta04910), and growth hormone (bta04935; Table 3) are related to major metabolic substrates (glucose and insulin) and their levels, rather than a direct effect on body energy homeostasis [70]. The major candidate genes (*GHSR, INS, INSR, LIPE, JAK1,* and *LEPR*) are directly associated with regulating these pathways. These metabolic responses act to keep metabolic homeostasis, leading to different feed efficiency by catabolic process, mainly energy mobilization and protein degradation [43, 45, 71]. The cAMP pathway (bta04024) affects a cascade that modulates numerous cellular events, including hormones, neurotransmitters, and other signaling molecules [72], associated with the regulation of lipolysis in adipocytes (bta04923) and can regulate the feed efficiency by mediating lipid metabolism. The activation of the cAMP pathway leads to the lipolysis of lipid droplets. In this context, Xu et al. [73] observed that the cAMP signaling pathway affects the feed efficiency of pigs through different mediations in lipid metabolism in the adipose tissues. Thus, the major genes (*GHSR, LIPE, ADCY3, PDE4B, PLD1, INSR, INS,* and *NPY*) related to cAMP and regulation of lipolysis in adipocytes lead to higher feed efficiency in animals by differences in lipid synthesis and degradation of the lipid content of adipocytes.

### Gene ontology (GO)

Gene enrichment showed the main biological mechanisms related to phenotypic divergence for feed efficiency-related traits (Supplementary Table S1 and S2). These biological processes highlight the effect on hormonal stimuli and signaling, which supports the relationship between feed intake and energy expenditure. We also found biological terms related to insulin and glucose levels, lipid metabolic process, precursor metabolites, energy metabolism, and muscle metabolism and contraction (Supplementary Table S1 and S2).

Biological processes affecting muscle metabolism and contraction (GO:0050881, GO:0090257, GO:0030048, GO:0006937, GO:0070252, GO:0003012, GO:0034103, GO:0048771, and GO:1901861; Supplementary Table S1) represent a striking effect for high feed efficiency contributing to the difference in energy expenditure by the skeletal muscle. Fu et al. [40] observed that ATP synthesis is comparatively lower in the skeletal muscle tissues in more efficient pigs. In contrast, Carvalho et al. [74] reported that differences in skeletal muscle energy expenditure contribute to Nellore cattle feed efficiency differences. In this context, biological processes affecting muscle metabolism and contraction suggest an important role in skeletal muscle homeostasis contributing to differences in FE-related traits in Nellore cattle [6]. In addition, biological processes associated with tissue remodeling (GO:0034103 and GO:0048771) and muscle development (GO:1901861) lead to differences in protein metabolism between efficient and inefficient animals. These complex biological processes controlling the muscle energy expenditure are known to play a key role in feed efficiency, in which animals with low RFI showed less protein degradation than those with high RFI [75]. Thus, lower protein turnover in more efficient animals can be an essential factor for lower muscle maintenance energy requirement, consequently reducing up 37% of energy expenditure [76].

Biological processes related to insulin and glucose levels, lipid metabolic process, and energy metabolism highlight the importance of metabolic signals leading to adjustments in feed intake and energy expenditure (Supplementary Table S1). These biological processes also provide a link between peripheral signals conveying information about energy homeostasis and hypothalamic signals associated with anabolic and catabolic pathways [71]. In addition, biological processes associated with hormonal stimuli and signaling, and modulation of the feeding intake rate (Supplementary Table S2), indicated a link between the hypothalamic signal and feed efficiency. Overall, integrating endocrine and metabolic modulators serves as a crucial neuroendocrine factor to control feed intake by adjusting feed intake and energy expenditure. Thus, the hypothalamus receives information about the animal's nutritional and metabolic status to regulate neuropeptides' expression, controlling the feed intake by anorexigenic (POMC, LEPR, LEPROT, and GHSR) and orexigenic (NPY) signals [47].

The enrichment pathway analyses complemented the multi-trait meta-analysis statistical test of GWAS results, contributing to unraveling the complexity of the FE-related traits control. In this framework, the FE-related traits were determined by combining different genomic regions (Fig. 1) and biological mechanisms (Supplementary Table S1 and S2) affecting physiological events with metabolic and endocrine signals changes, resulting in differences in feed intake and energy homeostasis. Thus, these results permit a better interpretation of the biological control of cattle feed efficiency through metabolic aspects and neural control mediating the catabolic and anabolic pathways, with effects on energy balance leading to specific physiological signals. Overall, the results obtained can be used to search for causal mutations or as a strategy to pre-select SNP markers for use in genomic selection approaches aiming to reduce the number of markers and calculation time and avoid overfitting the model [77, 78].

## Conclusion

The multi-trait meta-analysis statistical test of GWAS and enrichment analyses allowed the identification of key genomic regions with significant effects on neuroendocrine signals and energy homeostasis controlling feed efficiency-related traits in Nellore cattle. These genomic regions on BTA 1, 2, 3, 4, 7, 8, 11, 14, 15, 18, 21, and 29 surrounding genes related to biological mechanisms that regulate the whole-body level's energy balance with a striking effect for FE-related traits (ADG, FCR, RFI, DMI, and FE) across the Nellore cattle population. Thus, the pleiotropic effects of the key-regulatory genomic regions are directly implicated in the regulation of energy metabolism, and hypothalamus signaling may have an essential effect on FE-related traits in the two Nellore cattle populations. Overall, the major genomic regions uncovered across the Nellore cattle population are related to major modulators linking the metabolic homeostasis and genes that regulate the feeding intake allowing greater feed efficiency.

## Supplementary Information

**Additional file 1: Supplementary Table S1.** Functional enriched Biological Process (BP) related to metabolism among the candidate genes identified using the multi-trait meta-analysis statistical test for feed efficiency-related traits. **Supplementary Table S2.** Functional enriched Biological Process (BP) related to metabolism among the candidate genes identified using the multi-trait meta-analysis statistical test for feed efficiency-related traits. **Supplementary Figure S1.** Principal component analysis of animals based on the first two principal components based on SNP information to evaluate the extent of the population structure in the IZ population (NeC – Nelore Control; NeS – Nelore Selection and NeT – Nelore Traditional) and Qualitas population (QLT). **Supplementary Figure S2.** Manhattan plots of the percentage of the additive genetic variance explained by SNP-windows of 100 adjacent SNPs for average daily gain (ADG) and feed conversion rate (FCR) in *IZ and Qualitas (QLT) population*. **Supplementary Figure S3.** Manhattan plots of the percentage of the additive genetic variance explained by chromosome regions of 100 adjacent SNP windows for residual feed intake (RFI) and dry matter intake (DMI) in *IZ and Qualitas (QLT) population.***Supplementary Figure S4.** Manhattan plots of the percentage of the additive genetic variance explained by chromosome regions of 100 adjacent SNP windows for feed efficiency (FE) in the *IZ and Qualitas (QLT) population.*

## Data Availability

The data that support the findings of this study have belonged to commercial and experimental breeding programs, and restrictions are applied to the availability of these data, which were used under license for the current study, and so are not publicly available. However, data are available by contacting the corresponding authors upon reasonable request and with permission of Qualitas breeding program (https://qualitas.agr.br/) and experimental breeding program (contacting the researcher https://maria.mercadante@sp.gov.br.
