# Supplementary material for: Meta-analysis across Nellore cattle populations identifies common metabolic mechanisms that regulate feed efficiency-related traits
Source: BMC Genomics. 2022 Jun 7;23:424. doi: 10.1186/s12864-022-08671-w (PMC9172108; doi:10.1186/s12864-022-08671-w)
Supplement: Supplementary file 1 — Additional file 1: Supplementary Table S1. Functional enriched Biological Process (BP) related to metabolism among the candidate genes identified using the multi-trait meta-analysis statistical test for feed efficiency-related traits. Supplementary Table S2. Functional enriched Biological Process (BP) related to metabolism among the candidate genes identified using the multi-trait meta-analysis statistical test for feed efficiency-related traits. Supplementary Figure S1. Principal component analysis of animals based on the first two principal components based on SNP information to evaluate the extent of the population structure in the IZ population (NeC – Nelore Control; NeS – Nelore Selection and NeT – Nelore Traditional) and Qualitas population (QLT). Supplementary Figure S2. Manhattan plots of the percentage of the additive genetic variance explained by SNP-windows of 100 adjacent SNPs for average daily gain (ADG) and feed conversion rate (FCR) in IZ and Qualitas (QLT) population. Supplementary Figure S3. Manhattan plots of the percentage of the additive genetic variance explained by chromosome regions of 100 adjacent SNP windows for residual feed intake (RFI) and dry matter intake (DMI) in IZ and Qualitas (QLT) population.Supplementary Figure S4. Manhattan plots of the percentage of the additive genetic variance explained by chromosome regions of 100 adjacent SNP windows for feed efficiency (FE) in the IZ and Qualitas (QLT) population. [file 12864_2022_8671_MOESM1_ESM.docx]

**Meta-analysis across Nellore cattle populations identifies common metabolic mechanisms that regulate feed efficiency-related traits**

Lucio F. M. Mota^1*^, Samuel W. B. Santos^2^, Gerardo A. Fernandes Júnior^2^, Tiago Bresolin^1^, Maria E. Z. Mercadante^3,5^, Josineudson A. V. Silva^4,5^, Joslaine N. S. G. Cyrillo^3^, Fábio M. Monteiro^3^, Roberto Carvalheiro^2,5^, Lucia G. Albuquerque^2,5*^

^1^Department of Agronomy, Food, Natural Resources, Animals and Environment (DAFNAE), University of Padua, Legnaro - PD, 35020, Italy

^2^São Paulo State University (UNESP), School of Agricultural and Veterinarian Sciences, Jaboticabal - SP, 14884-900, Brazil.

^3^Institute of Animal Science, Beef Cattle Research Center, 14174-000 Sertãozinho – SP, Brazil

^4^São Paulo State University (UNESP), School of Veterinary Medicine and Animal Science, Botucatu – SP, 18618-681, Brazil

^5^National Council for Science and Technological Development, Brasilia - DF, 71605-001, Brazil

*Corresponding author:

Lucio F. M. Mota: [flaviommota.zoo@gmail.com](mailto:flaviommota.zoo@gmail.coma) and Lucia G. Albuquerque: [galvao.albuquerque@unesp.br](mailto:galvao.albuquerque@unesp.br)

# Supplementary material

Additional File 1: Supplementary Table S1. Functional enriched Biological Process (BP) related to metabolism among the candidate genes identified using the multi-trait meta-analysis statistical test for feed efficiency-related traits.

| ID | Description | p-value | q-value | geneID |
| --- | --- | --- | --- | --- |
| Muscle metabolism | | | | |
| GO:0050881 | musculoskeletal movement | 0.015420 | 0.038129 | ASCL1, TNNI2, TNNT3 |
| GO:0090257 | regulation of muscle system process | 0.000474 | 0.024551 | CNN1, GHSR, IGF1, IGFBP5, KCNQ1, PDE4B, TNNI2, TNNT3 |
| GO:0030048 | actin filament-based movement | 0.013363 | 0.037571 | KCNQ1, MYO7A, PDE4B, TNNI2, TNNT3 |
| GO:0006937 | regulation of muscle contraction | 0.004057 | 0.032922 | CNN1, GHSR, KCNQ1, PDE4B, TNNI2, TNNT3 |
| GO:0070252 | actin-mediated cell contraction | 0.026099 | 0.040181 | KCNQ1, PDE4B, TNNI2, TNNT3 |
| GO:0003012 | muscle system process | 0.026612 | 0.040257 | CNN1, GHSR, IGF1, IGFBP5, KCNQ1, PDE4B, TNNI2, TNNT3 |
| GO:0034103 | regulation of tissue remodeling | 0.046571 | 0.042440 | LEPR, CEACAM1, TGFB1 |
| GO:0048771 | tissue remodeling | 0.001298 | 0.028478 | IGFBP5, LEPR, ACP5, AXL, CEACAM1, TGFB1, RAB3D |
| GO:1901861 | regulation of muscle tissue development | 0.006564 | 0.034798 | IGF2, IGFBP5, TGFB1 |
| Insulin metabolism | | | | |
| GO:0032868 | response to insulin | 0.000014 | 0.003615 | AGRP, GHSR, HSD11B2, IGF1R, IGF2, INS, INSR, LPL, LYN, ATP6V1B2, RETN, CEACAM1, TNFSF10, ATP6V0D1 |
| GO:0048009 | insulin-like growth factor receptor signaling pathway | 0.000017 | 0.004883 | GHSR, IGF1, IGF1R, IGFBP2, IGFBP5 |
| GO:0043567 | regulation of insulin-like growth factor receptor signaling pathway | 0.000067 | 0.012084 | GHSR, IGF1, IGFBP2, IGFBP5 |
| GO:0043568 | positive regulation of insulin-like growth factor receptor signaling pathway | 0.000213 | 0.018198 | GHSR, IGF1, IGFBP5 |
| GO:0032869 | cellular response to insulin stimulus | 0.000279 | 0.019629 | GHSR, IGF1R, IGF2, INS, INSR, LPL, ATP6V1B2, CEACAM1, ATP6V0D1 |
| GO:0008286 | insulin receptor signaling pathway | 0.002257 | 0.030703 | IGF1R, IGF2, INS, INSR, ATP6V1B2, ATP6V0D1 |
| GO:1900076 | regulation of cellular response to insulin stimulus | 0.023799 | 0.043188 | IGF2, INS, LPL |

Additional File 1: Supplementary Table S1 (cont.). Functional enriched Biological Process (BP) related to metabolism among the candidate genes identified using the multi-trait meta-analysis statistical test for feed efficiency-related traits.

| ID | Description | p-value | q-value | geneID |
| --- | --- | --- | --- | --- |
| Glucose metabolism | | | | |
| GO:0042593 | glucose homeostasis | 0.000308 | 0.020144 | CYP7A1, IGF1R, IGFBP5, INS, INSR, LEPR, POMC, TH, STXBP5L |
| GO:0010907 | positive regulation of glucose metabolic process | 0.000567 | 0.023378 | IGF1, IGF2, INS, INSR |
| GO:0010906 | regulation of glucose metabolic process | 0.000881 | 0.025716 | IGF1, IGF2, INS, INSR, LEPR, POMC |
| GO:0046323 | glucose import | 0.002730 | 0.031712 | IGF1, INS, INSR, RAB4B |
| GO:0046326 | positive regulation of glucose import | 0.003300 | 0.032717 | IGF1, INS, INSR |
| GO:0006006 | glucose metabolic process | 0.008503 | 0.037733 | IGF1, IGF2, INS, INSR, LEPR, POMC |
| Lipid metabolism | | | | |
| GO:0045834 | positive regulation of lipid metabolic process | 0.000525 | 0.022973 | CYP7A1, GHSR, INS, LYN, SMPD3, TGFB1, CD81 |
| GO:0016042 | lipid catabolic process | 0.001374 | 0.028073 | PNPLA6, CYP7A1, PLA2G15, INS, LIPE, LPL, PLD1, ACER3, SMPD3, NCEH1 |
| GO:0019216 | regulation of lipid metabolic process | 0.001427 | 0.028272 | CYP7A1, GHSR, INS, LYN, SMPD3, ANGPTL8, CEACAM1, TGFB1, NCOA1, TGS1, CD81 |
| GO:0055088 | lipid homeostasis | 0.004181 | 0.033971 | CYP7A1, INS, LCAT, LPL, ANGPTL8 |
| GO:0006644 | phospholipid metabolic process | 0.017070 | 0.041427 | PNPLA6, EFR3B, PLA2G15, LCAT, LPL, PLD1, SMPD3, PLPPR2, ITPKC |
| GO:0042632 | cholesterol homeostasis | 0.011654 | 0.039404 | CYP7A1, LCAT, LPL |
| Energy metabolism | | | | |
| GO:0006112 | energy reserve metabolic process | 0.000164 | 0.016799 | IGF1, IGF2, INS, INSR, LEPR, POMC |
| GO:0043467 | regulation of generation of precursor metabolites and energy | 0.000770 | 0.025004 | AK4, IGF1, IGF2, INS, INSR, POMC, NUP37 |
| GO:0015980 | energy derivation by oxidation of organic compounds | 0.036477 | 0.045451 | IGF1, IGF2, INS, INSR, LEPR, POMC |
| GO:0006631 | fatty acid metabolic process | 0.003844 | 0.033525 | CYP2B6, CYP2F1, CYP7A1, PLA2G15, GHSR, CYP2S1, INS, LPL, CEACAM1, TH |
| GO:0019217 | regulation of fatty acid metabolic process | 0.013417 | 0.040150 | CYP7A1, GHSR, INS, CEACAM1 |

Additional File 1: Supplementary Table S2. Functional enriched Biological Process (BP) related to metabolism among the candidate genes identified using the multi-trait meta-analysis statistical test for feed efficiency-related traits.

| ID | Description | p-value | q-value | geneID |
| --- | --- | --- | --- | --- |
| Hormone signals | | | | |
| GO:0006836 | neurotransmitter transport | 0.013805 | 0.040302 | SLC18A1, STXBP2, TH, SYT8, STXBP5L |
| GO:0007218 | neuropeptide signaling pathway | 0.000930 | 0.026004 | AGRP, NPY, PENK, PMCH, POMC |
| GO:0001505 | regulation of neurotransmitter levels | 0.003719 | 0.033350 | PAH, SLC18A1, STXBP2, TH, SYT8, STXBP5L |
| GO:0002790 | peptide secretion | 0.004504 | 0.034365 | GHSR, IGF1, INS, PARD6A, SMPD3, TGFB1, LTBP4, STXBP5L, RAB3D |
| GO:0002791 | regulation of peptide secretion | 0.025789 | 0.043614 | GHSR, IGF1, INS, PARD6A, TGFB1, STXBP5L |
| GO:0045927 | positive regulation of growth | 0.003163 | 0.032493 | GHSR, IGF1, IGF2, INS, INSR, SPTBN4 |
| GO:0060416 | response to growth hormone | 0.000467 | 0.022353 | GHSR, IGFBP5, LYN, LEPROT |
| Feed intake control | | | | |
| GO:0007631 | feeding behavior | 0.000003 | 0.000279 | AGRP, GHSR, INS, LEPR, NPY, PMCH, RETN, TH |
| GO:0060259 | regulation of feeding behavior | 0.000003 | 0.000691 | AGRP, GHSR, INS, LEPR, RETN |
| GO:0031667 | response to nutrient levels | 0.000327 | 0.020460 | DNMT3A, KANK2, GHSR, HSD11B2, IGFBP2, LPL, ASCL1, NPY, PENK, POMC, TH, RIPOR1, NCOA1 |
| GO:0031670 | cellular response to nutrient | 0.000622 | 0.023870 | KANK2, LPL, PENK, NCOA1 |
| GO:0007584 | response to nutrient | 0.000922 | 0.025961 | DNMT3A, KANK2, IGFBP2, LPL, ASCL1, PENK, NCOA1 |


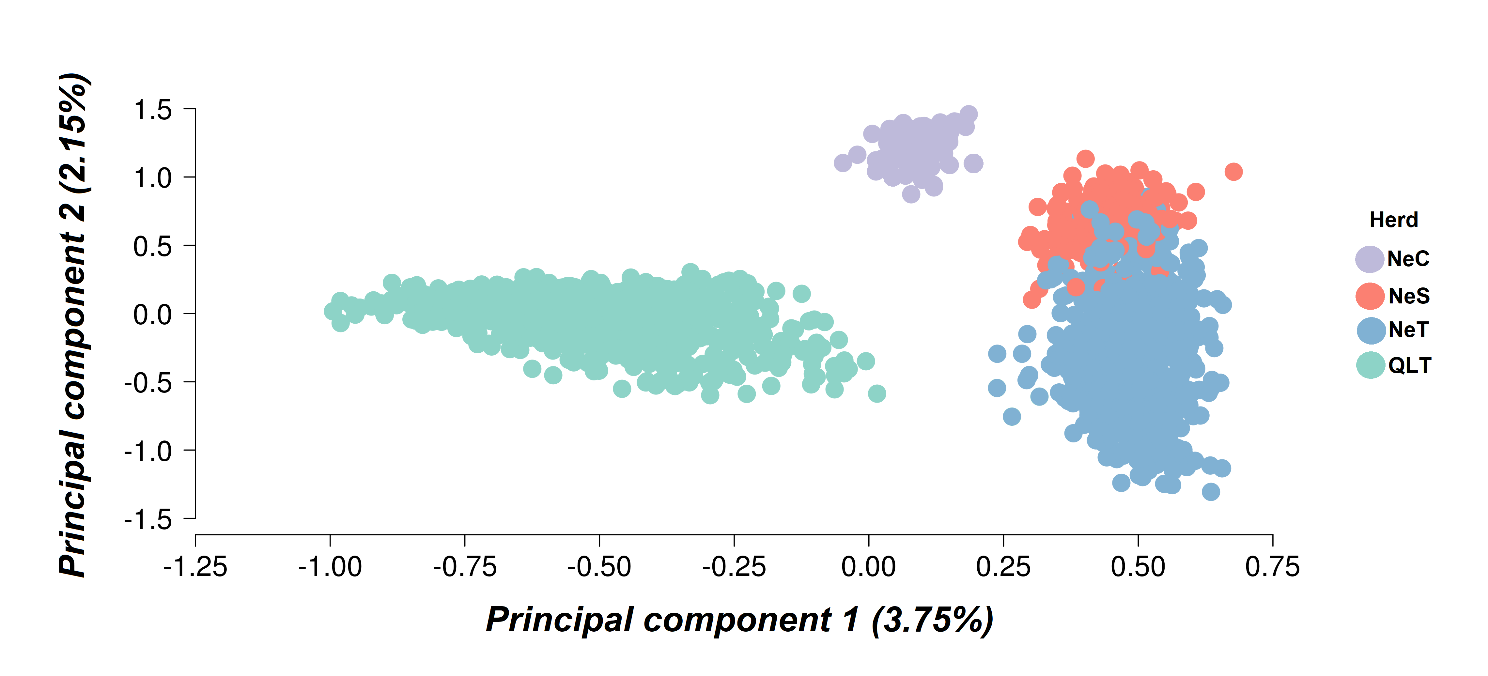


Additional File 1: Supplementary Figure S1. Principal component analysis of animals based on the first two principal components based on SNP information to evaluate the extent of the population structure in the IZ population (NeC – Nelore Control; NeS – Nelore Selection and NeT – Nelore Traditional) and Qualitas population (QLT).


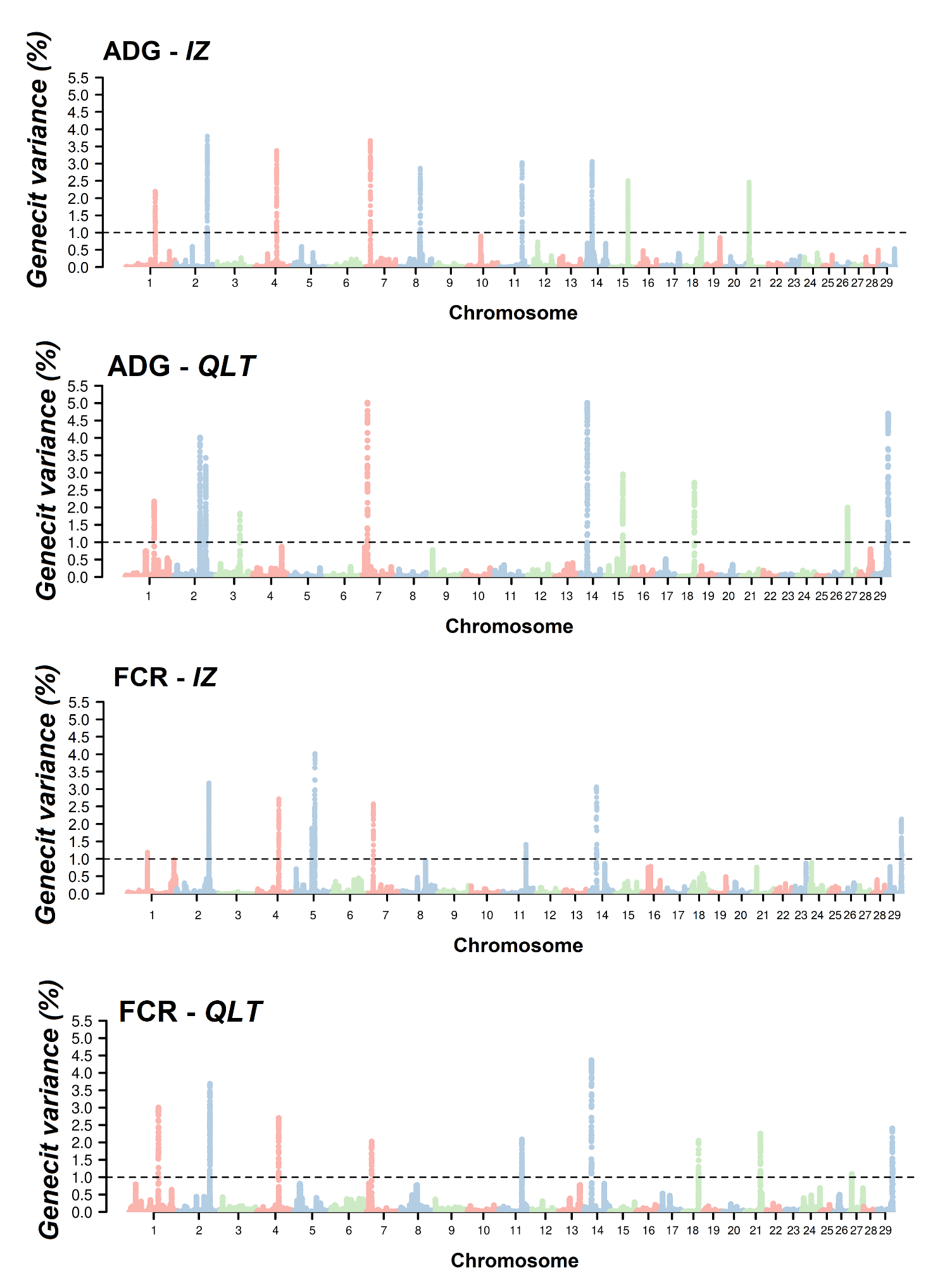


Additional File 1: Supplementary Figure S2. Manhattan plots of the percentage of the additive genetic variance explained by SNP-windows of 100 adjacent SNPs for average daily gain (ADG) and feed conversion rate (FCR) in *IZ and Qualitas (QLT) population*.


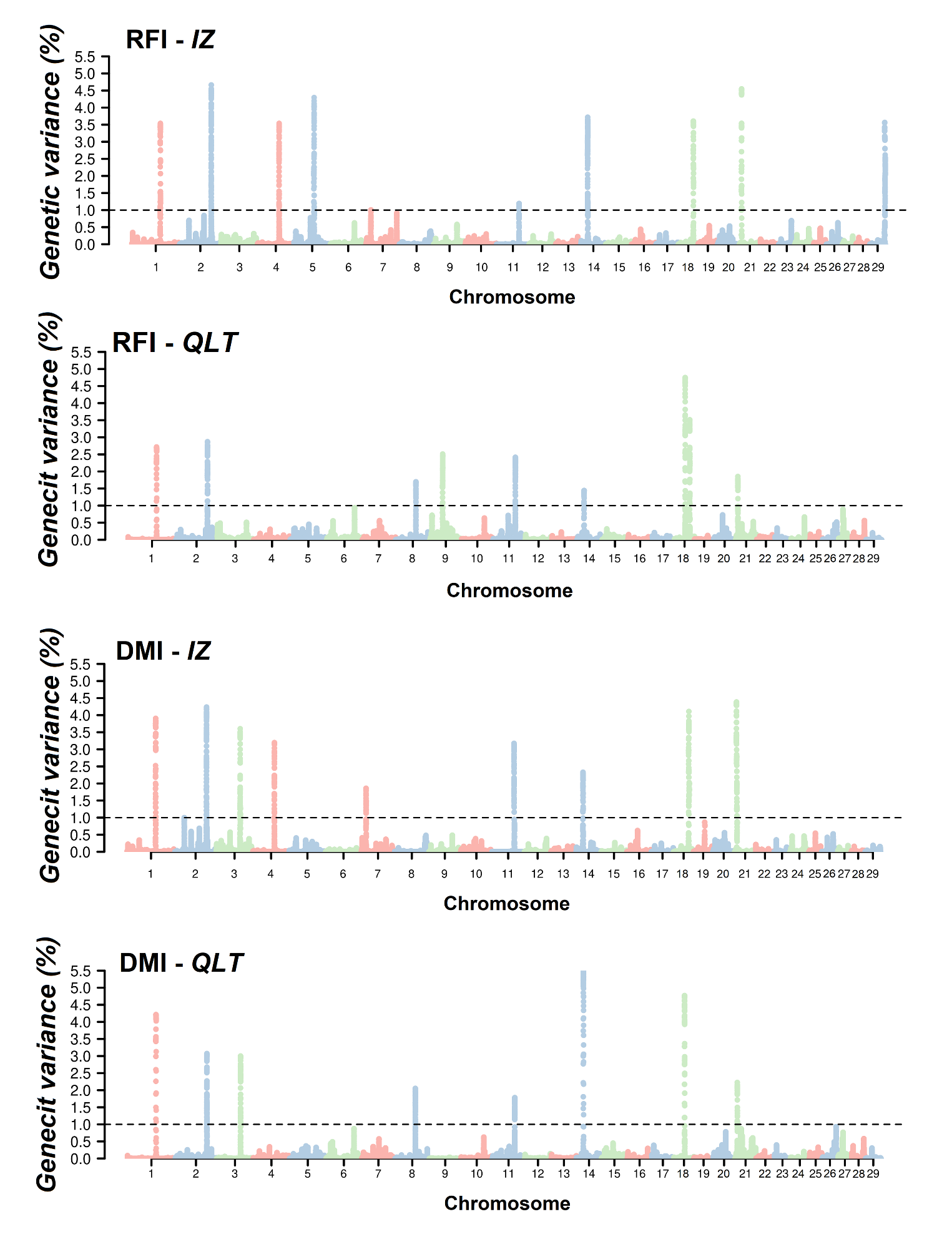


Additional File 1: Supplementary Figure S3. Manhattan plots of the percentage of the additive genetic variance explained by chromosome regions of 100 adjacent SNP windows for residual feed intake (RFI) and dry matter intake (DMI) in *IZ and Qualitas (QLT) population.*


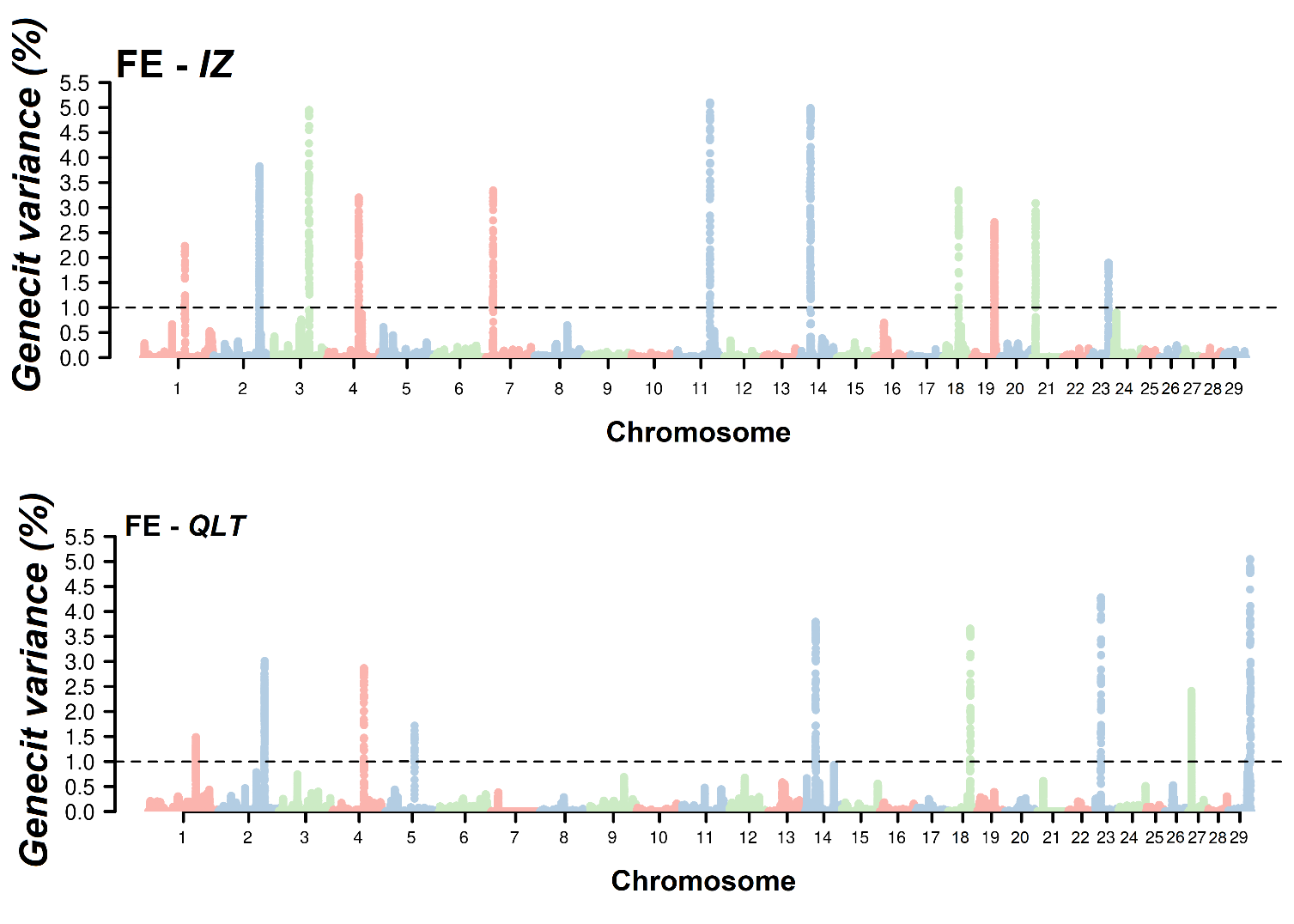


Additional File 1: Supplementary Figure S4. Manhattan plots of the percentage of the additive genetic variance explained by chromosome regions of 100 adjacent SNP windows for feed efficiency (FE) in the *IZ and Qualitas (QLT) population.*
